# Maternal and infantile gut mycobiome during the weaning period in free ranging Tibetan macaques (*Macaca thibetana*)

**DOI:** 10.1002/ece3.10108

**Published:** 2023-05-18

**Authors:** Ran Yue, Huijuan Chen, Xiaojuan Xu, Yingna Xia, Yu Sun, Mengyi Xia, Dongpo Xia, Binghua Sun

**Affiliations:** ^1^ School of Resource and Environmental Engineering Anhui University Hefei China; ^2^ International Collaborative Research Center for Huangshan Biodiversity and Tibetan Macaque Behavioral Ecology Anhui University Hefei China; ^3^ School of Life Sciences Hefei Normal University Hefei China; ^4^ School of Life Sciences Anhui University Hefei China

**Keywords:** gut mycobiome, Tibetan macaque, variation, weaning

## Abstract

Gut microbiome is critical to the health of mammals. Many previous studies have revealed the gut bacterial microbiomes of mother and infant changed significantly during the weaning period. However, little is known concerning the gut mycobiome of wild primates. Here, we examined the variations on gut mycobiome between weaning and post‐weaning for both mother and infant in wild‐living Tibetan macaques (*Macaca thibetana*). Our results showed that the gut mycobiomes of mother and infant were dominated by two phyla Ascomycota and Basidiomycota. For both mother and infant, the ASV richness of gut mycobiome remained relatively steady from weaning to post‐weaning periods, while the Shannon indexes increased significant in weaning compared to post‐weaning periods. However, no significant difference between mother and infant ASV richness and Shannon indexes during weaning and post‐weaning periods respectively. Compared to mothers, we found that much more known taxa of gut fungi were enriched in weaning or post‐weaning periods of infants. In particular, we found that the dominant genus *Aspergillus* was enriched in infants during weaning period. Furthermore, we found that the relative abundance of plant pathogens were significantly higher in the post‐weaning period than in the weaning period for infants. Our results indicated that weaning events could affect the gut mycobiome significantly for both mothers and infant in Tibetan macaques, which had a stronger effect on the gut mycobiome of infant monkeys than on their mothers.

## Graphical abstract

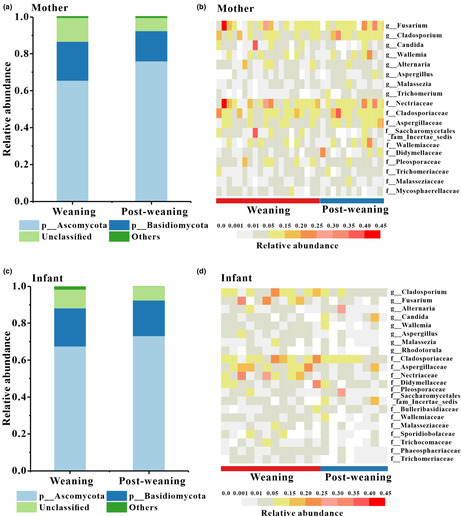

## 1 INTRODUCTION

Weaning is an important event in the development of mammals, involves a series of changes including physiology, nutrition and behavior (Guevarra et al., 2019; Waran et al., 2008). For example, there is a significant increase in gut pathogens when solid food is introduced after piglet weaned, which usually causes diarrhea in piglets (Li, Guo, et al., 2018). Horses have altered sleep patterns after weaning, increased glucocorticoid levels, decreased weight and performance, and increased risk of infectious diseases (Mach et al., 2017). After weaning, significant changes in the serum metabolome of mothers in Atlantic gray seal (*Halichoerus grypus*) affect their metabolic stability (Watson et al., 2020). In primates, maternal carrying and nursing decreases as weaning begins, and infants develop social behaviors and become increasingly socialized (Mach et al., 2017; Pavé et al., 2016).

Gut microbiome plays an important role in preventing pathogen colonization (Brestoff & Artis, 2013), absorbing nutrients (Rinninella et al., 2019) and regulating stress levels (Li et al., 2021). There is growing evidence that the composition and diversity of the gut microbial community changes with the weaning status of the infant and that these changes are of great benefit to the host in adapting to weaning and regulating health status. For example, studies on mammals, such as mergansers (*Cervus nippon*, Li, Wang, et al., 2018), humans (Amarri et al., 2006; Thompson et al., 2015; Guevarra et al., 2018; Wang et al., 2004), and piglets (Guevarra et al., 2018), have revealed that the composition and structure of the gut microbiome changed significantly when the first solid foods were introduced and gradually replaced the breast milk‐based diet. In addition, several studies on social animals such as rats (*Mus musculus*, Desbonnet et al., 2014), termites (*Zootermopsis angusticollis*, Rosengaus et al., 2011), hounds (*Crocuta crocuta*, Thaiss et al., 2016) and baboons (*Papio cynocephalus*, Tung et al., 2015) have found that social interactions can promote the spread of gut microbes and influence the composition and structure of individual gut microbiome. Moreover, there is growing evidence that the gastrointestinal tract responds to stress hormones by synthesizing cytokines, hormones, and neurotransmitters, which may alter microbiome diversity and increase pathogen colonization (Mach et al., 2017).

In general, the current researches on the gut microbiome of mammals during weaning period is mainly based on the bacterial community, little is known about the function and composition of the gut fungal community (also known as the gut mycobiome). Gut mycobiome also play a major role in consuming nutrients, facilitating nutrient extraction (Hoffmann et al., 2013) and regulating the immune system, forming a defense against harmful pathogens (Cui et al., 2013), and can even alter the composition of intestinal bacteria in the host (Huffnagle & Noverr, 2013). Recent study on piglet has showed a dramatic shift over weaning time in the bacterial and fungal communities, and an increase in network connectivity between the two kingdoms (Arfken et al., 2020). In humans, the mother's fungal community could transfer to their infant (Schei et al., 2017), and the fungal community changes significantly after the first year of life and influence in the infant's early immune development (Boutin et al., 2021). NHPs are important animal model systems for understanding many aspects of human physiology, health and the gut microbiome. However, the changes of gut mycobiome in the weaning period for both mother and infant remain to be clarified in wild primates.

In this study, we used high‐throughput sequencing to study the fecal mycobiome of free ranging Tibetan macaques (*Macaca thibetana*) at Mt. Huangshan, Anhui Province, China. The group named Yulinkeng 1 (YA1) has been monitored continuously since 1986. All individuals can be identified by specific physical characteristics such as body size, fur color, facial features, and body scars. Our behavioral team has well documented birth and weaning behaviors. We are primarily interested in exploring: (1) Whether there are significant differences in gut fungal diversity between weaning and post‐weaning periods both in mother and infant? (2) What are the differences in the composition and ecological function of the gut mycobiome between weaning and post‐weaning periods both in mother and infant? We present our results in the context of what is known about the fungi community in previous studies and discuss the potential impacts of weaning on the primate's gut mycobiome.

## 2 MATERIALS AND METHODS

### 2.1 Study site and subjects

This study was carried out at the Valley of Wild Monkeys (VWM) located in Mt. Huangshan National Reserve. The habitats are deciduous and evergreen broad‐leaf mixed montane forests. During our study period, the YA1 group of Tibetan macaques contained 58 individuals (7 infants, 11 adult males and 12 adult females), of which 9 adult mothers and 6 infants participated in this study. We have tracked and studied the group continuously for more than 30 years. The age of all individuals as well as their female kinship is clear, and each macaque could be followed to collect the behavioral data and fresh fecal samples.

In the present study, we observed the behavior of mothers and infants from the pre‐weaning (i.e., lactation) until they were in the post‐weaning period. The pre‐weaning period in Tibetan macaques is approximately 5–6 months, and we considered the first weaning conflict observed between mother and infant as the beginning of weaning, and the period from infant's birth to the beginning of weaning was defined as pre‐weaning. In addtion, the post‐weaning period was defined as 1 month after the last nipple contact. Due to the uncertainty in the field, it is difficult to collect samples from both mothers and infants at the same day. In particularly, fecal samples of infants are especially difficult to collect. All samples for this study were collected from July 20, 2021 until May 30, 2022. During this time, four students worked together to closely follow the monkeys to ensure that each fecal sample collected from a different individual. We obtained a total of 64 fresh fecal samples from Tibetan macaques, including pre‐weaning (3 fecal samples), weaning (41 fecal samples), post‐weaning (20 fecal samples) from mothers and infants. Due to the difficulty of collecting fecal samples of infants during pre‐weaning period, only 3 fecal samples were collected from infants in the pre‐weaning period, which belonged to two individual infants, so the pre‐weaning period was not included in this study. For this study, a total of 61 fecal samples (29 from mothers and 12 from infants) was analyzed. Samples information were listed in Table S1.

All stool samples were collected, stored and shipped at RNAlater (QIA‐GEN). Our stool samples were transported in time to the Anhui University laboratory in insulated boxes containing ice packs, and subsequently stored and preserved at −80°C until sent out for analysis. This research was approved by the Institutional Animal Care and Use Committee of the Anhui Zoological Society (permit number AHZS201711008). We performed all experiments in accordance with their approved guidelines and regulations, and complied with all principles of the China Animal Ethics Committee.

### 2.2 DNA extraction and sequencing

DNA was extracted from frozen stool samples using a QIAamp DNA Stool Mini Kit (Qiagen, Inc., Valencia CA), following the manufacturer's protocol with a bead‐beating procedure. Total DNA extracted from 61 stool samples was sent to the Shanghai Majorbio Bio‐pharm Technology Co., Ltd. for analysis. The ITS regions were identified by ITS1F (5′‐CTTGGTCATTTAGAGGAAGTAA‐3′) and ITS2 (2043R; 5′‐GCTGCGTTCTTCATCGATGC‐3′) primers (Bokulich & Mills, 2013). PCR reaction mixtures contained 5–100 ng of DNA template, 1 × GoTaq Green master mix, 1 M MgCl_2_, and 5 pmol of each primer. The ITS regions were identified by ITS1F (5′‐CTTGGTCATTTAGAGGAAGTAA‐3′) and ITS2 (2043R; 5′‐GCTGCGTTCTTCATCGATGC‐3′) primers (Bokulich et al., 2013). PCR reaction mixtures contained 5–100 ng of DNA template, 1 × GoTaq Green master mix, 1 M MgCl_2_, and 5 pmol of each primer.

### 2.3 Data analysis

We trimmed raw FASTQ sequencing data for the adaptor sequence and for quality control using the sliding window approach implemented in fastp (v0.19.6) (Chen et al., 2018). A window of 50 bp was set to filter the reads with a tail mass value of 20 or less. If the average mass value in the window was lower than 20, the rear bases were removed from the window, and the reads with a tail mass value of 50 bp after quality control were filtered. Those containing N bases were removed. We merged overlapping paired‐end reads using FLASH (v1.2.7) (Magoc & Salzberg, 2011), with the minimum overlap set to 10 bp, the maximum error ratio of overlap area was 0.2, and the number of mismatches barcode allowed was 0. The maximum primer mismatch number was 2. Lastly, we clustered the quality‐check of sequences into Amplicon Sequence Variants (ASVs) using DADA2 within Qiime 2 to truncate forward and reverse reads, to denoise the data, and to detect and remove chimeras (Bolyen et al., 2019; Callahan et al., 2016). Naive Bayes Classifier was used for taxonomic identification of the ASV sequences, and BLAST searches were conducted using Unite databases (Deshpande et al., 2016).

The Shannon diversity index (Shannon), ASV richness, and unweighted and weighted UniFrac distance matrices were calculated using Qiime 2 (Bolyen et al., 2019). The FUNGuild v1.0 database, 2 an open annotation tool for parsing fungal community datasets by ecological guild, was used to assign ecological functions to each ASV (Nguyen et al., 2016). During this analysis, we only considered all assignments with a confidence score of “probable” or “highly probable”. Genera not represented in the database or with a confidence score of “possible” were classified as undetermined.

We tested for normal distributions in alpha diversity indices, relative abundances of dominant phyla, and functional guilds using the Kolmogorov–Smirnov normality test. Principal coordinates analysis (PCoA) was performed with the R packages Made4 and Vegan. Permutational multivariate ANOVA (PERMANOVA) was used to test for differences in beta diversity (unweighted and weighted UniFrac distance) using the Adonis function in the vegan R package (Chen et al., 2012). Linear discriminant analysis effect size (LEfSe) was used with default options to determine genera enriched in each study group (Segata et al., 2011). In all analyses, the value of *p* was set at .05.

## 3 RESULTS

### 3.1 General patterns of the fungal profile

**FIGURE 1:**
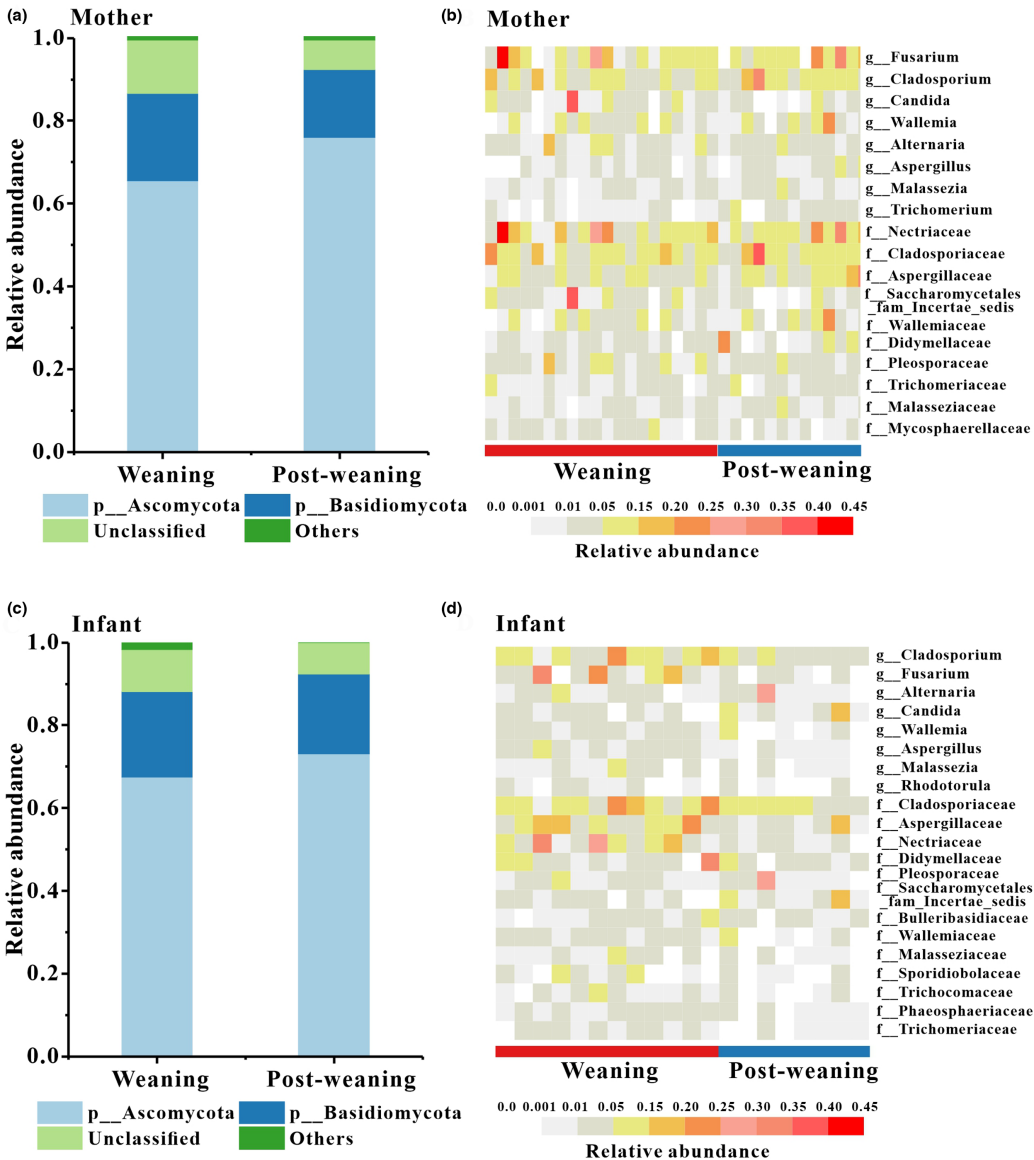
The distributions of phylum, families, and genera. (a) Relative abundance of mothers' fecal fungal taxa at the phylum level. (b) The distributions of core families and genera of fecal fungi in mothers. (c) Relative abundance of infant' fecal fungal taxa at the phylum level. (d) The distributions of core families and genera of fecal fungi in infant. (a, c) Stacked bar graphs illustrate the abundances of phyla, and the *x*‐axis represents the sample groups. (b, d) Core families and genera were defined as average relative abundance >0.01 and present in more than 90% of all fecal samples.

We obtained 2,397,029 high‐quality filtered reads. To eliminate the effects of different sequencing depth on the analyses, the data set was rarefied to 23,138 sequences per sample (the minimum sequence number among 61 samples). Taxonomic assignment revealed 12 phyla, 47 classes, 133 orders, 341 families, 821 genera and 7233 ASVs. The dominant phyla across all samples were Ascomycota (*x* = mean ± SD, *x* = 68.91 ± 32.09%) and Basidiomycota (*x* = 19.85 ± 27.78%; Figure 1a,c). At the family level, mother group's fecal samples were dominated by Nectriaceae (Mother: *x* = 11.41 ± 32.51%) and Cladosporiaceae (Mother: *x* = 8.91 ± 27.30%). Infant group's fecal samples were dominated by Cladosporiaceae (Infant: *x* = 8.40 ± 15.41%) and Aspergillaceae (Infant: *x* = 7.21 ± 17.68%). The dominant genera for all samples were *Fusarium* (Mother: *x* = 10.51 ± 30.08%, Infant: *x* = 5.45 ± 27.16%) and *Cladosporium* (Mother: *x* = 8.10 ± 25.44%, Infant: *x* = 6.30 ± 15.95%).

We defined core genera and families as being present in more than 90% of fecal samples with an average relative abundance >1%. At the family level, 10 and 13 core families were detected in mother and infant groups respectively. (Figure 1b). Nine core families were shared by mother and infant, which are Nectriaceae, Cladosporiaceae, Aspergillaceae, Didymellaceae, Pleosporaceae, Wallemiaceae, Saccharomycetales_fam_Incertae_sedis, Trichomeriaceae and Malasseziaceae. One (Mycosphaerellaceae) and four (Bulleribasidiaceae, Sporidiobolaceae, Phaeosphaeriaceae and Trichomeriaceae) core families only were detected in the maternal and infant groups, respectively. At the genus level, there are 8 core genera including (*Fusarium*, *Cladosporium*, *Candida*, *Wallemia*, *Alternaria*, *Aspergillus*, *Malassezia and Trichomerium*) were detected in the mother sample group. Similarly, 8 core genera *Fusarium*, *Cladosporium*, *Candida*, *Wallemia*, *Alternaria*, *Aspergillus*, *Malassezia and Rhodotorula* were present in the infant sample groups (Figure 1d).

### 3.2 Diversity and composition of the gut mycobiome

**FIGURE 2:**
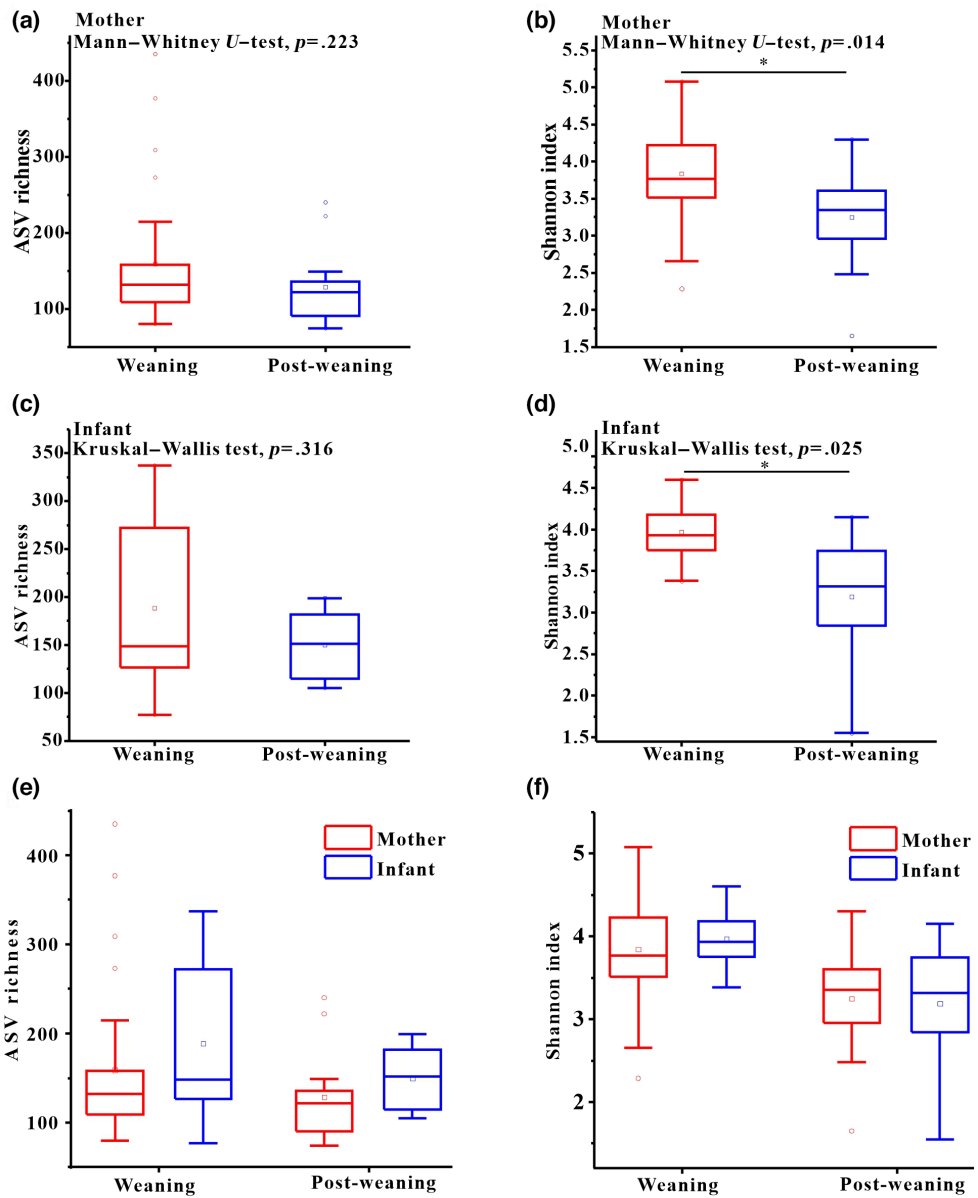
Differences in fecal fungal alpha diversity between mother and infant groups. (a) Comparison of ASV richness in mother groups. (b) Comparison of Shannon diversity indexes in mother groups. (c) Comparison of ASV richness in infant groups. (d) Comparison of Shannon diversity indexes in infant groups. (e) Comparison of ASV richness between mother and infant groups. (f) Comparison of Shannon diversity indexes between mother and infant groups. Mann–Whitney *U* test for pairwise comparison tests. *p < .05; **p < .01, ***p < .001.

By comparing the alpha diversity of maternal gut mycobiome including ASV richness and Shannon index, we found that there were not significantly different of ASV richness between weaning and post‐weaning of mothers (Mann–Whitney *U*, *p* = .223; Figure 2a), but the Shannon index of mothers was significantly higher in weaning period than that in post‐weaning (*p* = .014; Figure 2b). Similarly results also were detected in infants, the ASV richness of infants did not show significant differences between weaning and post‐weaning periods (*p* = .316; Figure 2c), while the Shannon index of weaning period was significantly higher than that in post‐weaning period (*p* = .025; Figure 2d). Moreover, we compared the ASV richness and Shannon index between mother and infant at same period including weaning and later post‐weaning. The results showed that there was no significant difference between mother and infant ASV richness during weaning and post‐weaning periods respectively (Mann–Whitney *U*, weaning, *p* = .187; post‐weaning, *p* = .245; Figure 2e), the results of Shannon index showed no significant difference (weaning, *p* = .456; post‐weaning, *p* = .877; Figure 2f).

**FIGURE 3:**
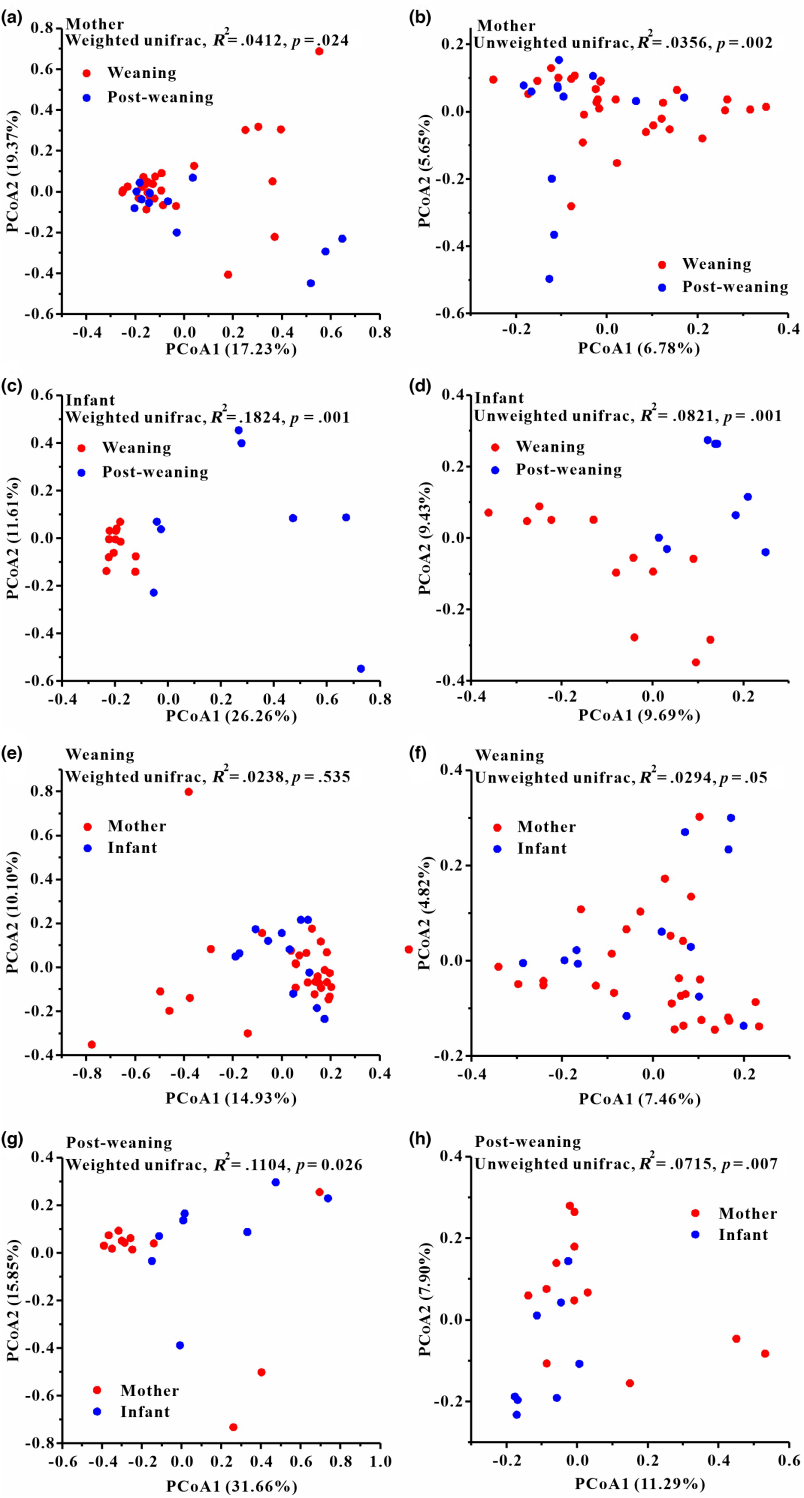
Differentiation of fecal fungal beta diversity between mother and infant groups. (a, b) Differences in mother group between weaning and post‐weaning, (a) based on unweighted UniFrac distances and (b) weighted UniFrac distances. (c, d) Differences in infant group between weaning and post‐weaning, (c) based on unweighted UniFrac distances and (d) weighted UniFrac distances. (e, f) Differences in weaning period between mother and infant, (e) based on unweighted UniFrac distances and (f) weighted UniFrac distances. (g, h) Differences in post‐weaning period between mother and infant, (g) based on unweighted UniFrac distances and (h) weighted UniFrac distances. Principal coordinates analysis (PCoA) was used to show patterns across two study groups. Adonis tests were performed on unweighted and weighted UniFrac, respectively. Significance was set at the 0.05 level.

We performed PCoA and PERMANOVA tests based on unweighted and weighted unifrac dissimilarities to investigate the variation of beta diversity in the mycobiome across all samples. Our results showed significant variation in the overall gut mycobiome composition of the weaning and post‐weaning samples in mothers (weighted, *F* = 1.676, *R* ^2^ = .0412, *p* = .024; unweighted, *F* = 1.4378, *R* ^2^ = .0356, *p* = .002; Figure 3a,b). Similarly, the distribution of overall gut mycobiome was significantly different between the weaning and post‐weaning samples in infants (weighted, *F* = 4.0151, *R* ^2^ = .1824, *p* = .001; unweighted, *F* = 1.611, *R* ^2^ = .0821, *p* = .001; Figure 3c,d). In addition, there was no significant difference in fecal mycobiome composition of mother and infant samples during weaning period. (weighted, *F* = 0.952, *R* ^2^ = .0238, *p* = .535; unweighted, *F* = 1.1803, *R* ^2^ = .029, *p* = .05; Figure 3e,f). However, there was significant difference in fecal mycobiome composition of mother and infant samples during post‐weaning period (weighted, *F* = 2.2334, *R* ^2^ = .1104, *p* = .026; unweighted, *F* = 1.3871, *R* ^2^ = .0715, *p* = .007; Figure 3g,h).

**FIGURE 4:**
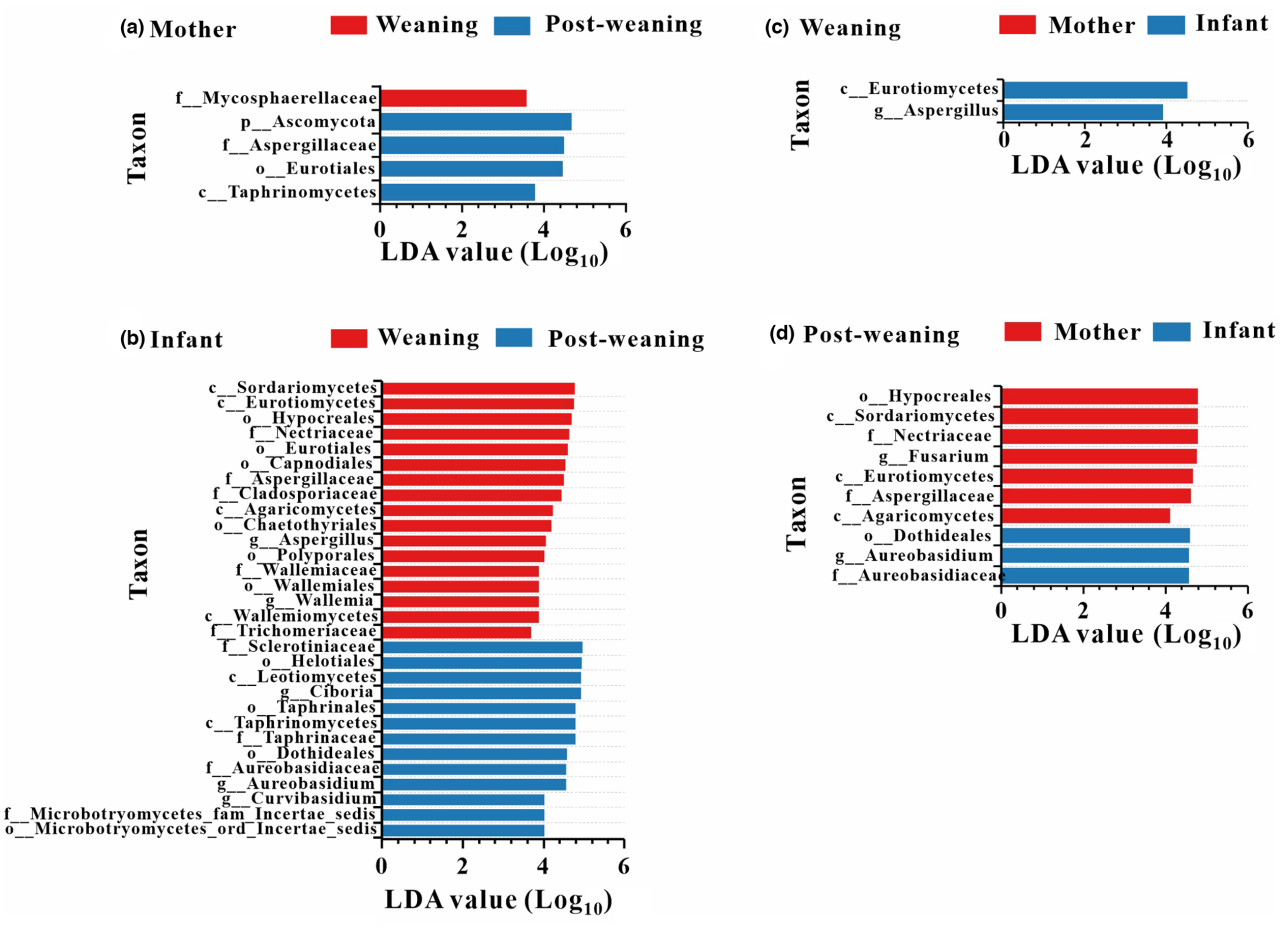
Indicators of known fungal taxa in weaning and post‐weaning periods. (a) Indicators of known fungal taxa of weaning and post‐weaning in mother group. (b) Indicators of known fungal taxa of weaning and post‐weaning in infant group. (c) Indicators of known fungal taxa of mother and infant during weaning period. (d) Indicators of known fungal taxa of mother and infant during post‐weaning period. Gut microbial taxonomy enriched in different sample groups identified by LEfSe (LDA > 3, *p* < .05).

Linear discriminant analysis effect size analyses showed that each study group were characterized by different known fungal taxa (at the genus, family, order, class, and phylum levels; the mean relative abundance of known taxa accounting for ≥1% of all the fecal samples). In mothers, four known taxonomic units were enriched at post‐weaning, and one known taxonomic units were enriched at weaning (LDA > 3, *p* < .05; Figure 4a). In contrast, a total of 30 known taxonomic units were significantly enriched in one of the two groups of infants (LDA > 3, *p* < .05). Among these taxa 17 and 13 were found in the weaning and post‐weaning respectively (Figure 4b). We defined a high occurrence indicator as being present in more than 90% of the stool samples in its corresponding group. In the mothers, only one family Mycosphaerellaceae was overrepresented in weaning periods, while three indicators, including phylum Ascomycota, order Eurotiales and family Aspergillaceae, were overrepresented in post‐weaning periods. For the infants, 15 occurrence indicators were overrepresented in weaning periods, including three classes (Wallemiomycetes, Agaricomycetes and Eurotiomycetes), five orders (Wallemiales, Chaetothyriales, Capnodiales, Eurotiales and Hypocreales), five families (Trichomeriaceae, Wallemiaceae, Cladosporiaceae, Aspergillaceae and Nectriaceae), and two genera (*Aspergillus* and *Wallemia*). In addition, one class (Leotiomycetes), two orders (Dothideales and Helotiales) were overrepresented in post‐weaning periods of infants.

In addition, we found that each study group (mother and infant) were characterized by different known fungal taxa during the same period (LDA > 3, *p* < .05). We found that only two known taxonomic units are enriched in infants during weaning periods (Figure 4c). In contrast, a total of 10 known taxonomic units were significantly enriched in post‐weaning mothers or infants. Among these taxa, seven and three were found in the mothers and infants respectively during the period of post‐weaning (Figure 4d). We defined a high occurrence indicator as being present in more than 90% of the stool samples in its corresponding group. In the samples of weaning periods, only one class Eurotiomycetes and one genus *Aspergillus* were overrepresented in infants. For post‐weaning periods, seven occurrence indicators were overrepresented in mothers, including three classes (Sordariomycetes, Eurotiomycetes, Agaricomycetes), one order Hypocreales, two families (Nectriaceae and Aspergillaceae), one genera *Fusarium*. In addition, order Dothideales, family Aureobasidiaceae and genera *Aureobasidium* were overrepresented in infants of post‐weaning periods.

### 3.3 Functional trophic modes of gut mycobiome

**FIGURE 5:**
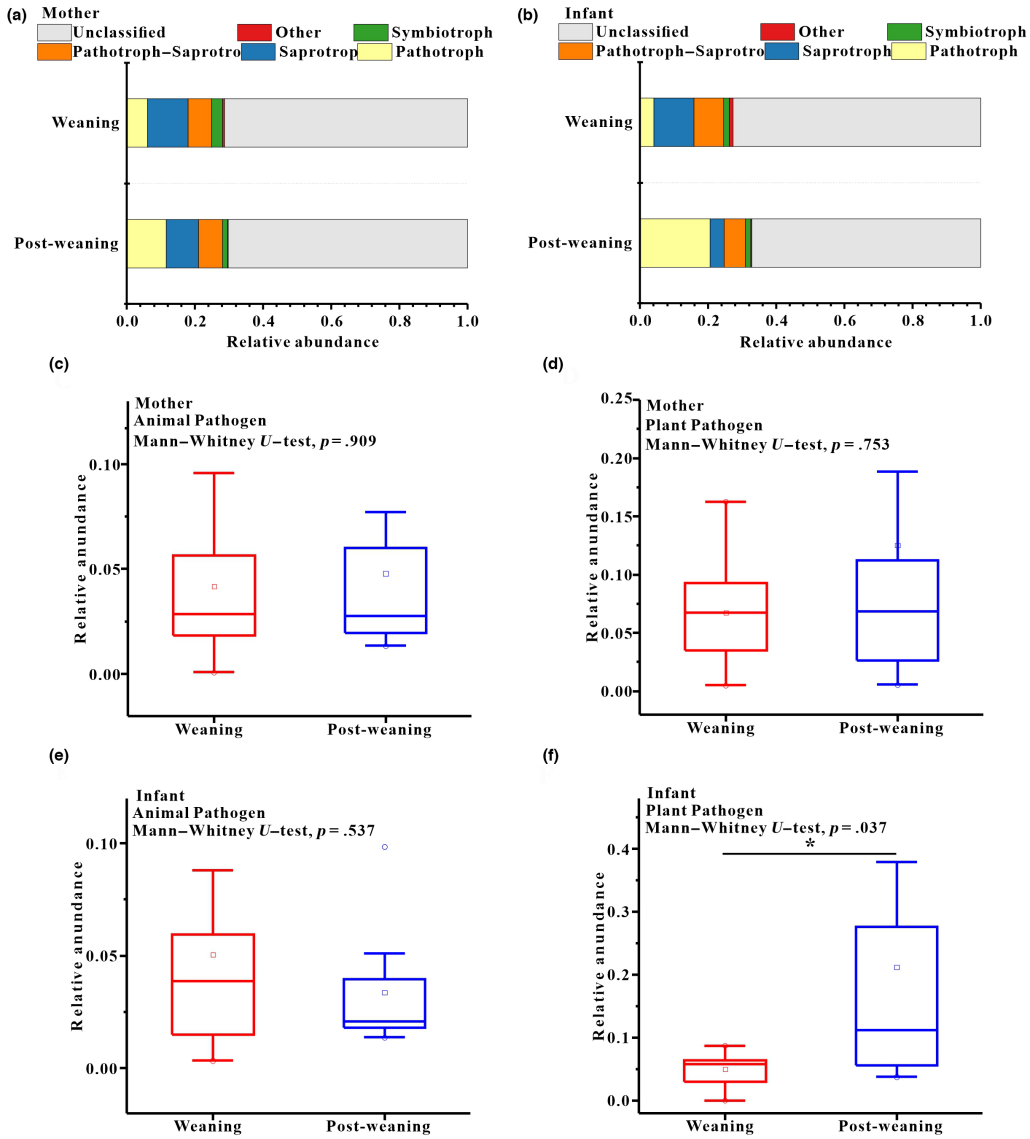
Variation of of gut mycobiome in functional trophic modes between infant and mother study groups. (a) Relative abundance of functional trophic modes in infants. (b) Relative abundance of functional trophic modes in mothers. Stacked bar graphs illustrate the abundances, *x*‐axis represents the sample types. (c, d) Comparison of the functional guilds of animal pathogens and plant pathogens in mothers. (e, f) Comparison of the functional guilds of animal pathogens and plant pathogens in infants. Mann–Whitney *U* test was used to evaluate the variation between treatment groups. *p < .05; **p < .01, ***p < .001.

Based on the FUNGuild v1.0 tool, we analyzed six functional trophic modes of fecal fungal communities in mothers and infants. The representative and dominant predictive functional trophic modes in all samples of mothers and infants were Saprotroph (*x* = mean ± SD, mother: *x* = 11.18 ± 18.40%; infant: *x* = 8.78 ± 10.72%), Pathotroph (mother: *x* = 7.67 ± 66.19%; infant: *x* = 10.71 ± 66.08%), Pathotroph‐Saprotroph (mother: *x* = 6.98 ± 21.03%; infant: *x* = 7.67 ± 13.44%), and Symbiotroph (mother: *x* = 2.73 ± 29.27%; infant: *x* = 1.56 ± 8.01%) (Figure 5a,b). In addition, two major fungi guilds (mean relative abundance was greater than 1%) were detected, namely animal pathogens (mother: *x* = 4.36 ± 15.32%; infant: *x* = 4.37 ± 14.58%) and plant pathogens (mother: *x* = 8.42 ± 66.38%; infant: *x* = 11.45 ± 65.62%). We compared the two major guilds (plant pathogens and animal pathogens) between weaning and post‐weaning periods in mothers and infants respectively. Our results showed that there were no significant differences between weaning and post‐weaning periods in mothers for these two guilds (Mann–Whitney *U*, animal pathogens, *p* = .909; plant pathogens, *p* = .753) (Figure 5c,d). Similarly, no significant differences in the relative abundance of animal pathogens between weaning and post‐weaning periods for infants (*p* = .537) (Figure 5e). However, the relative abundance of plant pathogens was significantly higher in the post‐weaning period than in the weaning period for infants (*p* = .037) (Figure 5f).

## 4 DISCUSSION

In current study, our data showed that maternal and infantile gut mycobiomes were dominated by two phyla Ascomycota and Basidiomycota both in the weaning and post‐weaning periods. This result is consistent with previous studies on humans and NHPs (Hallen‐Adams & Suhr, 2017; Strati et al., 2016; Binghua Sun et al., 2018). Even though we did detect eight core genera in mother and infant sample groups respectively, seven of these core genera were shared by the two sample groups. In addition, we also found that there were no significant differences of ASV richness and Shannon indexes between mother and infant during weaning and post‐weaning periods respectively. All of these evidences indicated that infants of Tibetan macaque have initially established a gut mycobiome similar to those of adult females from weaning period. However, there was significant difference in fecal mycobiome composition of mother and infant samples during post‐weaning period.

Weaning, and the resulting dietary and physiological changes that occur in developing period, is thought to drive the establishment of gut microbiome in mammals (Rey et al., 2014; Williams et al., 2018). Our results showed that the richness of infants gut mycobiome remained relatively steady from weaning to post‐weaning periods, while their Shannon diversity increased significant in weaning compared to post‐weaning periods. This result is consistent with previous study in piglets (Arfken et al., 2020). In addition, the Shannon diversity of mothers during weaning also increased significantly compared to post weaning. Similarly, the significant differentiation in beta diversities of the weaning and post‐weaning samples were detected both in mothers and infants Tibetan macaques. As a necessary part of the biology of mammals for both mothers and infants, weaning is a combination of stressful events associated with physiological changes such as the increase in cortisol and catecholamines (Otten et al., 2010). It has been reported that the increase in stressor‐induced cortisol is associated with disruption of the gut microbiome including a dramatic increase in alpha diversity (Uren Webster et al., 2020). We speculate that stress and physiological fluctuations may result in the increase of alpha diversity in mother and infant during weaning. Moreover, healthy people generally have a low diversity of fecal mycobiome (Nash et al., 2017; Raimondi et al., 2019), while increased of fungal diversity has been linked to gastrointestinal disease (Kühbacher et al., 2006; Ott et al., 2008). We hypothesize that the same may be true in Tibetan macaques. To clarify the key factors affecting gut fungal diversity of Tibetan macaques during weaning, future studies that integrate the data onto hormones, diet and behavior are needed.

In addition, we found that much more known taxa of gut fungi were enriched in weaning or post‐weaning periods of infants compared those been detected in mothers. This result implied that the event of weaning had a stronger effect on the gut mycobiome of infant monkeys than on their mothers. Compared to adult female NHPs, the infants involve drastic changes in diet, physiology, gut microbiota and environment in the early development stage. All these is important factors affecting the instability of gut mycobiome (Dong & Gupta, 2019; Krüger et al., 2019; Mims et al., 2021). In particular, we found that the genus *Aspergillus* was enriched in infants during weaning period. *Aspergillus* has negative inferred interactions with beneficial bacteria like *Prevotella* which also have been reported as a core genus of bacterial microbiome in Tibetan macaques (Arfken et al., 2020; Sun et al., 2020). In addition, a negative correlation between short chain fatty acids (SCFAs) and *Aspergillus* has been reported in human and piglet studies (Arfken et al., 2020; Hoffmann et al., 2013). Our results together with previous evidences suggest that overrepresented of *Aspergillus* in weaning periods of infants may be detrimental to the development of their gut bacterial microbiome and health.

Moreover, our result showed the relative abundance of plant pathogens were significantly higher in the post‐weaning period than in the weaning period for infants. The most typical feature of the end of weaning for infant primates is a shift in dietary patterns of completely independent gain diet of the living environment. Our study group inhabits deciduous and evergreen broad‐leaf mixed montane forests. The main diets of Tibetan macaques consist of leaves, fruits, grass, and to a lesser extent, flowers and roots (Li & Kappeler, 2020). Environmental fungi are one of the main sources of gut mycobiome in mammals, especially the fungi carried by food resources. Our previous study has showed that the plant pathogen was dominant fungal guilds of gut mycobiome in Tibetan macaques (Sun, Xia, Davison, et al., 2021; Sun, Xia, Garber, et al., 2021), and plants diets possibly contributing more to seeding the macaque's gut mycobiome than soil fungi (Sun, Xia, Davison, et al., 2021; Sun, Xia, Garber, et al., 2021). We speculate that the increased in plant pathogens in the post‐weaning period of infant Tibetan macaques should be due to their completely plant diet like the adult individuals. In addition, given the infants are exposed to the soil with a month after birth, fungi from soil may affect the gut mycobiome of pre‐weaning infants. Affected by the difficulty of sample collection, Our current data are difficult to assess the role of environment fungi in the early establishment of gut mycobiome in infant monkeys.

## AUTHOR CONTRIBUTIONS

**Ran Yue:** Data curation (equal); formal analysis (equal); investigation (equal); methodology (equal); resources (equal); software (equal); visualization (equal); writing – original draft (equal); writing – review and editing (equal). **Huijuan Chen:** Data curation (equal); investigation (equal); resources (equal); software (equal); visualization (equal); writing – original draft (equal); writing – review and editing (equal). **Xiaojuan Xu:** Data curation (equal); formal analysis (equal); methodology (equal); project administration (equal); software (equal); writing – original draft (equal); writing – review and editing (equal). **Yingna Xia:** Data curation (equal); formal analysis (equal); resources (equal); software (equal); writing – original draft (equal); writing – review and editing (equal). **Mengyi Xia:** Investigation (equal); resources (equal); visualization (equal); writing – original draft (equal); writing – review and editing (equal). **Yu Sun:** Investigation (equal); methodology (equal); resources (equal); visualization (equal); writing – review and editing (equal). **Dongpo Xia:** Formal analysis (equal); methodology (equal); resources (equal); writing – original draft (equal); writing – review and editing (equal). **Binghua Sun:** Conceptualization (equal); data curation (equal); formal analysis (equal); funding acquisition (equal); methodology (equal); project administration (equal); supervision (equal); visualization (equal); writing – original draft (equal); writing – review and editing (equal).

## FUNDING INFORMATION

This project was supported by grants from the National Natural Science Foundation of China (grant nos. 31870371, 32171488) and Natural Science Foundation of Universities of Anhui Province (grant nos. KJ2021A0922).

## CONFLICT OF INTEREST STATEMENT

We have no conflict of interest to declare.

## Supporting information


Table S1

## Data Availability

Raw data were submitted to the Sequence Read Archive of NCBI under the accession number PRJNA933095.
