# Supplementary material for: Maternal and infantile gut mycobiome during the weaning period in free ranging Tibetan macaques (Macaca thibetana)
Source: Ecol Evol. 2023 May 18;13(5):e10108. doi: 10.1002/ece3.10108 (PMC10196218; doi:10.1002/ece3.10108)
Supplement: Supplementary file 1 — Table S1 [file ECE3-13-e10108-s001.docx]

Maternal and infantile gut mycobiome during the weaning period in free ranging Tibetan Macaques (*Macaca thibetana*)

- **Supplementary Material**

Ran Yue^1, 2^, Huijuan Chen^1, 2^, Xiaojuan Xu^3^*, Yingna Xia^1, 2^, Yu Sun^1, 2^, Mengyi Xia^1, 2^, Dongpo Xia^4^, Binghua Sun^1, 2^*

***Table S1*** Samples information used in this study. In the same row, two individuals are mother-child relationships.

| **Mothers** | | |  | **Infants** | | |
| --- | --- | --- | --- | --- | --- | --- |
| **Animals ID** | **Sampling number (Weaning)** | **Sampling number (Post-weaning)** |  | **Animals ID** | **Sampling number (Weaning)** | **Sampling number (Post-weaning)** |
| TXH | 4 | 1 |  | TQT | 4 | 1 |
| YCL | 4 | 1 |  | YXT | 1 | 1 |
| YCH | 3 | 0 |  | YXL | 2 | 0 |
| TXX | 5 | 1 |  | TQW | 2 | 1 |
| YXX | 3 | 1 |  | YQB | 0 | 0 |
| YXY | 3 | 1 |  | YQQ | 2 | 2 |
| THX | 3 | 4 |  | TFC | 0 | 0 |
| THY | 3 | 3 |  | TFY | 0 | 0 |
| YCY | 1 | 0 |  | YXC | 1 | 3 |
| Total = 61 | 29 | 12 |  |  | 12 | 8 |
